# Necrotizing Fasciitis: An Emergency Medicine Simulation Scenario

**DOI:** 10.7759/cureus.758

**Published:** 2016-08-31

**Authors:** Henrik Galust, Matthew H Oliverio, Daniel J Giorgio, Alexis M Espinal, Rami Ahmed

**Affiliations:** 1 Northeast Ohio Medical University; 2 Ohio University Heritage College of Osteopathic Medicine; 3 The Ohio State University; 4 Emergency Medicine, Summa Akron City Hospital, Summa Health System

**Keywords:** necrotizing fasciitis, emergency medicine, simulation, patient advocacy, interdisciplinary communication, simulation scenario

## Abstract

Necrotizing fasciitis (NF) is a rare and rapidly progressing life-threatening infectious process. By progressing through a simulation involving a patient with NF and participating in a post-scenario debriefing, learners will gain the necessary skills and knowledge to properly diagnose and manage patients with NF. Learners are taught to initiate appropriate and timely treatment and to advocate on behalf of their patient after inappropriate pushback from consultants to improve outcomes.

## Introduction

Necrotizing fasciitis (NF) is a rare and life-threatening infectious process. It is characterized by the rapid, yet often subtle, spread of inflammation and necrosis across fascial and subcutaneous tissues, leading to systemic signs of toxicity and pervasive tissue destruction and resulting in high morbidity and mortality [1]. Currently, the US Center for Disease Control and Prevention estimates that 500 to 800 cases of NF are identified in the United States annually [2]. Despite recent advances and improvements in the medical management of these patients, the case fatality rate of NF remains between 11% and 76% [2-4]. Risks and predisposing factors for NF include extremes in age, diabetes mellitus, peripheral vascular disease, immunocompromised states, and skin injury from surgery, procedures, burns, blunt trauma, childbirth, or IV drug use [4-7].

A high index of clinical suspicion with early recognition and appropriate treatment is vital for the prognosis of NF considering its fulminant progression. However, a timely and accurate clinical diagnosis is difficult, given the relative rarity of this disease and the paucity of early pathognomonic features [8]. Therefore, any patient presenting with inflammatory skin changes and additional symptoms of fever, toxic appearance, crepitation, pain out of proportion to the exam, blisters or bullae, and/or rapid clinical deterioration should be evaluated for NF [9]. Laboratory findings of appreciable leukocytosis and elevated serum creatine kinase (CK) levels, along with radiographic evidence of subcutaneous gas, should raise high suspicion for NF [5]. Surgical exploration remains the gold standard for definitive diagnosis and treatment of NF and should never be delayed, especially in the presence of subcutaneous emphysema or rapidly evolving soft tissue infection [1]. Studies have shown that delaying surgical intervention by as little as 24 hours increased patient mortality by as much as 20% [6].

Unfortunately, misdiagnosis of NF is common and mismanagement often leads to catastrophic outcomes [8]. Literature reviews suggest that 41% to 96% of cases of NF are falsely identified as less serious soft tissue complications, such as abscesses or cellulitis [8]. Failure to treat NF can lead to amputations, sepsis, multi-organ failure, and death [6]. Therefore, accurate and timely diagnosis and treatment are imperative; yet, the rate of misdiagnosis suggests that many clinicians have limited knowledge and experience in the appropriate evaluation and management of NF. This simulation scenario aims to provide an opportunity for health professionals to broaden their skill set and management of NF as a high-risk low-frequency presentation. This simulation will serve to guide clinicians to more rapidly and accurately diagnose NF and to initiate timely and aggressive management for improved patient outcomes.

## Technical report

**Figure 1:**
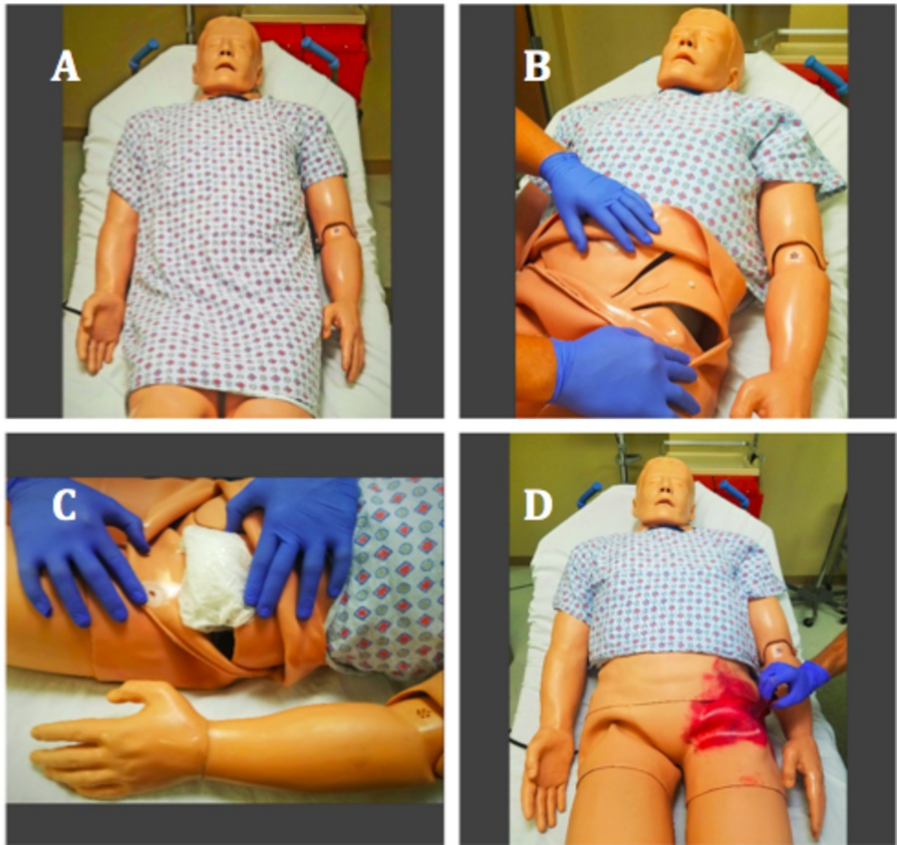
Preparing the simulator

The case for NF is conducted in a setting made similar to a moderate size community emergency department, with access to standard medications, diagnostic studies, and consultants. The laboratory is outfitted with standard intubation and central line equipment, a monitor, crash cart with a defibrillator as well as a high-fidelity simulator, Simulaids© Stat Manikin With Deluxe Airway Management Head (Simulaids, Inc., Saugerties, NY). The hip of the manikin is prepared with a standard moulage technique and a simple reversible modification to the simulator (Figure 1D) to give the appearance of erythema and ecchymosis as well as provide the feel of subcutaneous emphysema on palpation of the simulator skin, as manifested in some patients with NF [9]. Wax paper sheets or similar material are condensed and placed in the hip of the manikin to simulate the feel of subcutaneous emphysema upon palpation (Fig 1C).

### Replication of subcutaneous emphysema in the hip region

Materials Included: 1.     Approximately 2 feet of wax paper 2.    Simulaids© Stat Manikin With Deluxe Airway Management Head simulator 3.    Disposable gloves 4.    Moulage makeup to simulate blunt trauma/laceration

Application: 1.     Expose simulator hip region 2.    Add desired amount of wax paper to subcutaneous region of simulator 3.    Use moulage makeup to imitate erythema and tissue necrosis

### Preparing the simulation scenario:

**Figure 2:**
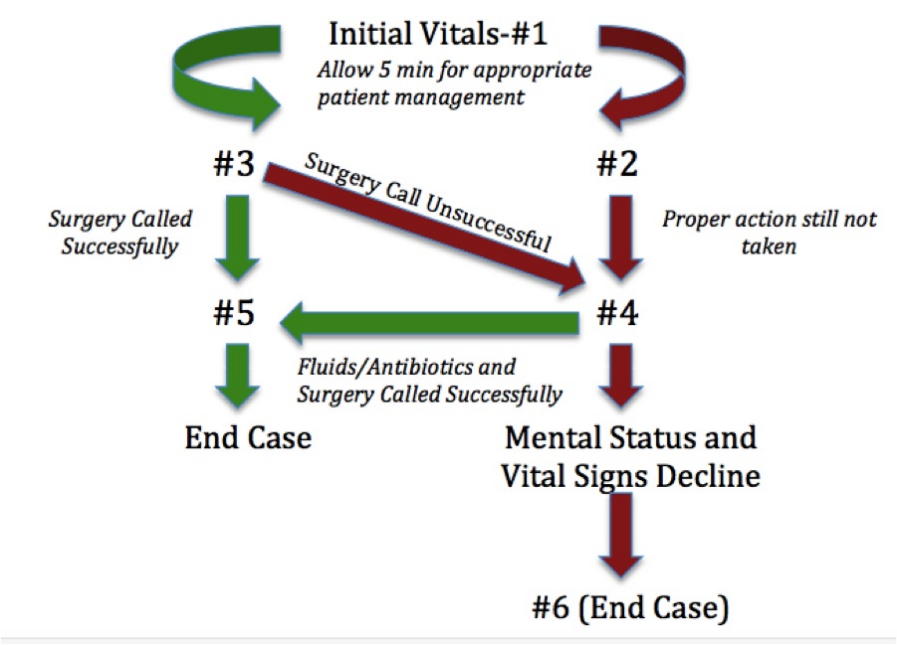
Vitals flow chart (branch points). *Numbers in figure correlate with vitals listed in Table 1.

Staff involved in the simulation includes a confederate scribe, a confederate nurse, a simulation technician, and at least one faculty member/content expert. Faculty are pre-briefed on the case and provided with a comprehensive case outline prior to simulation, which includes patient information, branch points, and vitals (Figure 2, Table 1). The manikin’s vitals are adjusted in real-time as outlined by the case branch points (Figure 2) and in response to the decisions of the learner (green arrows represent the correct pathway and red arrows represent the incorrect pathway).

**Table 1:** Initial Vital Signs

	Heart Rate	Blood Pressure	Temperature	O_2_ Sats (RA)	RR
#1	117	105/68	38.5	93%	18
#2	122	93/64	38.5	93%	18
#3	105	117/82	38	93%	18
#4	125	75/37	38	92%	20
#5	97	119/85	38	93%	16
#6	130	70/33	38	92%	22

**Figure 3:**
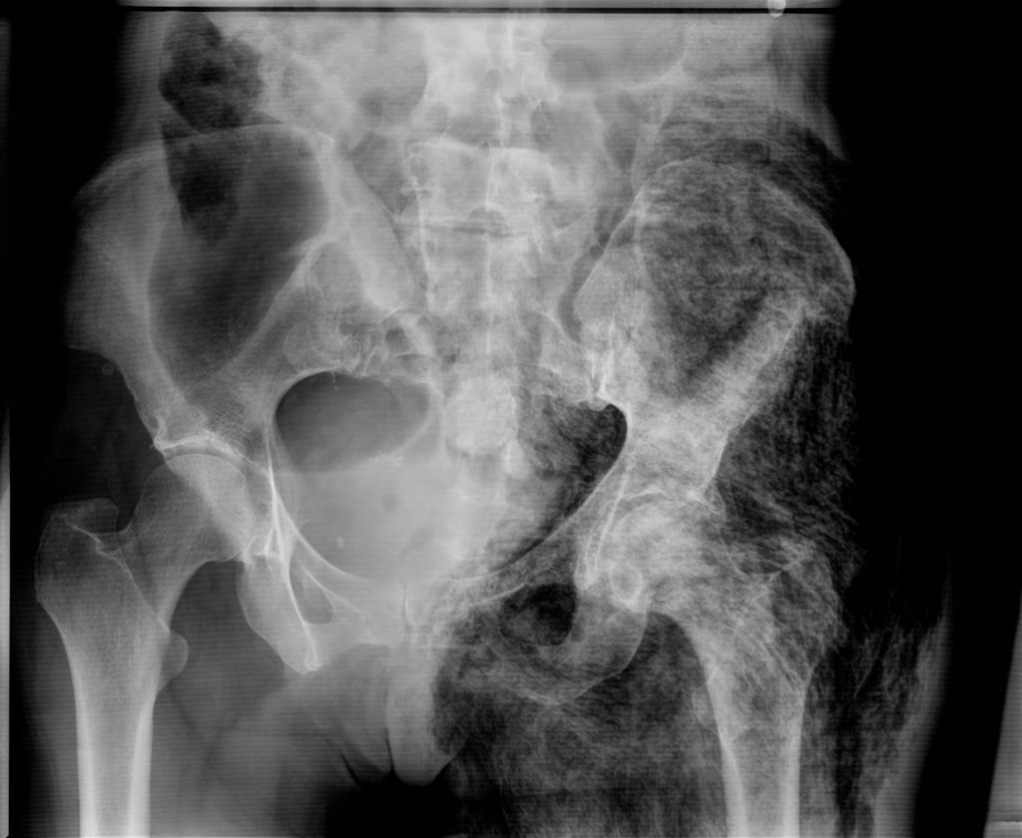
Anteroposterior pelvic X-ray highlighting diffuse linear lucencies of the left hemipelvis indicative of subcutaneous air along the fascial planes Courtesy of http://radiopaedia.org/cases/necrotising-fasciitis-1

**Figure 4:**
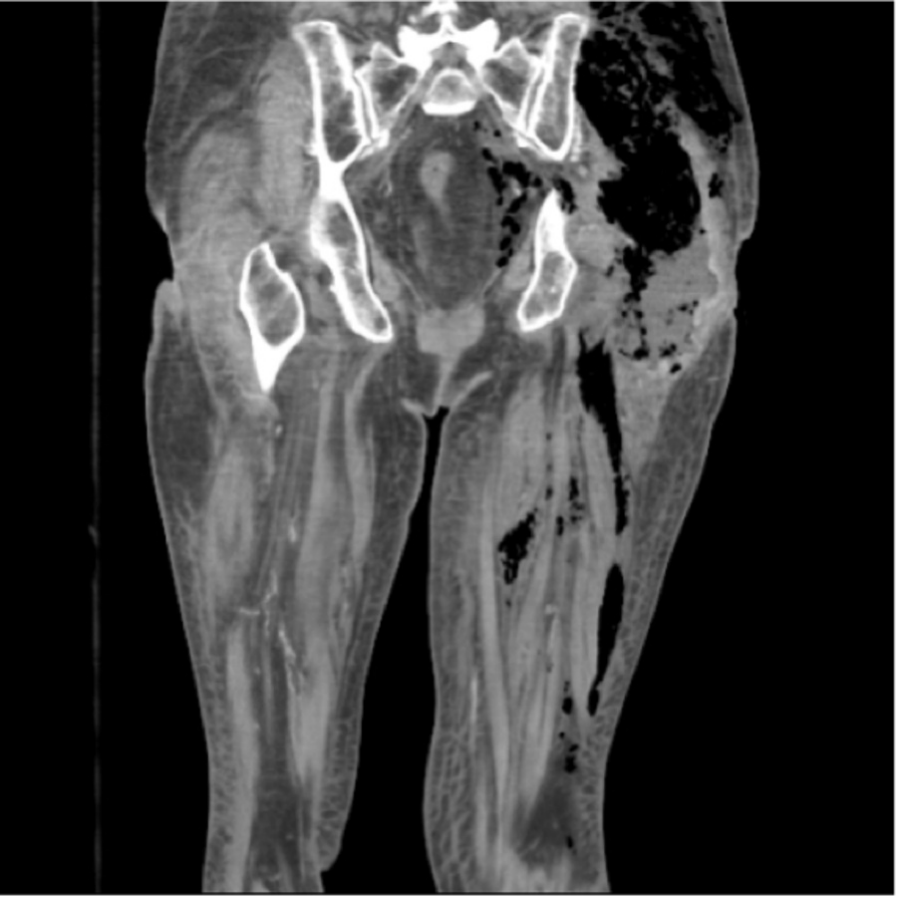
Frontal view of CT Pelvis & Lower extremities demonstrating subcutaneous gas tracking along fascial planes of left leg and into hemipelvis Courtesy of https://emrems.com/2013/04/06/necrotizing-fasciitis-2/

**Figure 5:**
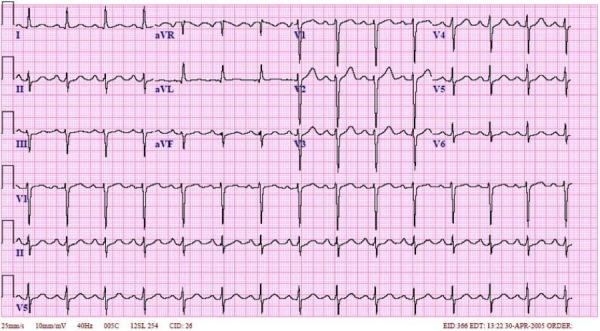
12 lead ECG (Normal Sinus Rhythm) Courtesy of http://interpretation.my3gb.com/ecg-interpretation.html

Image studies (Figures 3-4), 12-lead ECG (Figure 5), and pertinent labs (Table 2) are included and reviewed by faculty. A technician provides voice-over through the manikin to simulate patient verbal feedback. Details of the case are standardized and include the history of the present illness, prescription medications, and lab work ordered by the learners. A confederate nurse is present to execute orders and to relay information to learners upon request as the case progresses. 

**Table 2:** Lab Results

Complete Blood Count	Basic Metabolic Panel	Liver Function Test	Lactic Acid	Creatine Kinase	Arterial Blood Gas
WBC: 17,000 *10^3^/microL (4-11,000)	Sodium: 140 mEq/L (136-145)	Within normal limits	4.7 mg/dL (0-1.5)	>600 (0-4.9 ng/mL)	pH: 7.39 (7.35-7.45)
Hb: 12 mmol/dL (12-15)	Potassium:4 mEq/L (3.5-4.1)				pCO2: 40 mmHg (35-45)
	Chloride: 100 mmol/L (98-107)				
Hct: 35 mmol/dL (36-45)	CO2: 25 mmol/L (22-30)				pO2: 80 mmHg (60-100)
	BUN: 18 mg/dL (6-20)				
PLT: 175 *103/microL (100-250)	Creatinine: 1.5 mg/dL (0.6-1.4)				HCO3: 25 mEq/L (24-28)
	Glucose: 164 mg/dL (70-100)				

### Pre-briefing

Prior to the scenario, a pre-briefing session was held. The learners were informed that they should address the simulation as realistically as possible and were reminded of the importance of the fiction contract involving all simulation sessions. The high-fidelity manikin's limitations and capabilities were discussed, along with the availability of resources and roles of the staff during the simulation case (confederate nurse, technician, etc.) Lastly, learners discussed their individual roles amongst themselves prior to the initiation of the simulation.    

### Case 

In this simulation scenario, a 68-year-old female is brought in from home by paramedics, complaining of pain in her left hip. The EMS report provided by the confederate nurse states that the patient fell the previous day while doing yard work, making her less ambulatory. The learner encounters a patient complaining of left hip pain after a fall, nausea, and an inability to ambulate. She denies any head injuries or loss of consciousness. Percocet left over from past knee surgeries was taken and found to have no effect on her pain levels. The patient has a past medical history of diabetes, hypertension, chronic obstructive pulmonary disease (COPD), and peripheral vascular disease (PVD). Patient reports allergies to iodine and codeine. She reports that she is a pack-a-day smoker and has not used recreational drugs since using cannabis in college.

The case may be terminated if the learner has appropriately obtained a complete history and physical examination, noticed crepitus in the left hip, ordered appropriate imaging studies, began fluid/antibiotic resuscitation, and has called the surgical consultant who agrees to emergently evaluate the patient. This case was designed specifically to test the learner’s conviction when the consultant (inappropriately) advises that the patient can be treated with antibiotics and admitted to the medical team for further treatment. This scenario can be modified to include multiple acceptable procedures depending on the learner’s level of training (intubation, central venous access, etc). 

### Debriefing

After the case, a debriefing session was immediately held for all learners. The faculty-led discussion was facilitated through an advocacy and inquiry debriefing technique [10]. Whenever possible, faculty attempted to have learners self-identify and analyze their communication and leadership strategies, their working differential diagnosis, and their management decisions. Faculty encouraged learners to self-identify aspects managed successfully, as well as potential improvements to their performance. 

The main points of discussion include the standard management of the case (physical examination, proper imaging, antibiotics, etc.) as well as the role of the physician to advocate for his or her patient when inappropriate pushback (suggesting admission to medicine with intravenous antibiotics and re-assessment in several hours) from surgical consultants occurs. The ability to advocate for the patient to get an immediate evaluation by surgery or to quickly transfer the patient to definitive care at a tertiary care center was a key objective in the debriefing. 

Another prevalent topic of discussion revolved around the concept of physician “anchoring” and the potentially devastating consequences to the patient. In our experience with this case, on several occasions, novice physicians obtained a focused history and physical exam and prematurely created a very small differential diagnosis (pelvic fracture versus cellulitis) [11]. Due to the common occurrence of fractures as a result blunt trauma in the elderly, learners many times overlooked the obvious subcutaneous emphysema on physical exam and gas present on x-rays and initiated empiric treatment for an unstable pelvic fracture, given the hypotension and tachycardia. On several occasions, a pelvic binder was placed on the patient despite the x-rays demonstrating no obvious fracture. Emphasis was placed on the varying presentations of NF and the importance of a comprehensive clinical evaluation, including an evaluation of the skin, the character of the pain, and the general appearance of the patient. 

### Post-scenario didactics

After debriefing was completed, a focused 15-minute didactic session was implemented to ensure that the learners reinforce the key information and skills learned during the simulation. Emphasis was placed on key points related to identifying and treating necrotizing fasciitis. Topics regarding the diagnosis of NF included recognition of symptoms, such as subcutaneous emphysema and gas in the fascial planes seen on radiographs. The discussion of treatment stressed the importance of aggressive fluid and antibiotic administration and immediate surgical evaluation. In addition to the short lecture, time was allotted for learners to ask questions. 

## Discussion

Necrotising fasciitis is a rare medical condition that requires a high clinical index of suspicion with early intervention due to its serious and often fatal course. Therefore, it is imperative for clinicians to include this pathologic process in their differential diagnoses. Knowledge of the appropriate initial treatment and definitive care is vital for optimizing patient outcomes. Unfortunately, misdiagnosis of NF remains significantly high with as many as three out of four cases erroneously identified as other more prevalent and benign conditions [8]. Due to the relative rarity of this disease, many clinicians are lacking the necessary knowledge and experience to reach a timely diagnosis. Simulation cases are indispensable in providing this experience, offering a safe place to learn without the risks of morbidity and mortality that are inherent to NF. Our simulation allows for a particularly engaging, yet controlled, environment to challenge learners with all the nuances inherent to managing NF. We have structured our simulation to mirror an authentic presentation of NF as closely as possible through extensive consideration and planning.

The case was designed with the specific intent of raising the learner’s awareness of such an elusive condition. Successful resolution of the scenario hinges upon an accurate recognition of the key case findings. For example, proper diagnosis requires learners to demonstrate knowledge of major predisposing factors of NF, such as a history of diabetes mellitus and recent trauma. Learners then have to contextualize this information with additional pathognomonic findings, such as systemic signs of infection, subcutaneous emphysema on palpation, and radiographic evidence of soft tissue gas. 

In our past experience, failure by learners to arrive at an accurate diagnosis during these cases has been largely due to three contributing factors: lack of a sufficiently broad differential diagnoses that includes NF, overlooking key distinguishing findings (subcutaneous emphysema on palpation or gas on diagnostic imaging), or anchoring on an incorrect diagnosis (fixation error), leading to complete mismanagement of the simulated patient. During debriefing sessions, learners who demonstrated difficulty during this case consistently reported that focusing on narrow yet more commonly encountered etiologies (e.g. pelvic fracture or cellulitis) prevented them from recognizing the seriousness of the case and from ultimately arriving at a correct diagnosis. In several instances, learners admitted to reaching a level of certainty with their final diagnosis and were unable or unwilling to reconsider other diagnoses, even in light of contradicting evidence (fever, no improvement with pain medications). For example, many learners erroneously diagnosed and continued to maintain the diagnosis of pelvic fracture with hemorrhagic shock despite being provided with radiographic imaging that showed no acute fractures (yet grossly demonstrated air tracking through fascial planes). This type of diagnostic error, when medical practitioners make faulty conclusions by seeking out only confirming evidence even in the face of conflicting information, has been well described in the literature as a “confirmation” or “ascertainment” bias [12]. Several groups became so fixated on their identified diagnoses that they placed pelvic binders on patients and consulted orthopedic surgery for continued management. Learners admitted that they were so engrossed in their estimation of an acute hip fracture that they failed to recognize overt indicators of infection, such as fever, leukocytosis, and lactic acidosis. Such “tunnel vision” or “anchoring” errors are among the most commonly encountered medical diagnostic errors and are seen when medical practitioners focus on only a few clinical findings at the expense of fully appreciating the overall clinical picture [11]. In high-risk low-frequency presentations such as NF, such errors in heuristics can result in devastating outcomes for patients.

In addition to reaching an accurate diagnosis, another key objective of our simulation is for learners to develop the ability to appropriately and expediently treat NF. Learners must successfully demonstrate an understanding that the initiation of aggressive fluid resuscitation and empirical antibiotics are vital during the acute management of NF. Equally significant is taking early steps to confer with surgical specialists for both definitive diagnosis and therapeutic debridement.

One element we have chosen to include in this scenario is the addition of a degree of contention between the learner and surgical interventionalist/consultant. During the simulation, the learners request to speak with the on-call surgical specialist. During the conversation regarding the case management, the learner is met with initial hesitation and even resistance from the surgical consultant. For example, upon hearing the case, the surgical consultant repeatedly questions the certainty of the diagnosis of NF and states that the patient should rather “just be provided broad spectrum antibiotics overnight and admitted to the medical team.” In order to overcome this challenge, the learner is forced to assertively advocate on behalf of the patient, knowing the time-sensitive and critical nature of the underlying pathological state. This challenging social interaction is meant to emulate real life clinical situations where learners might be forced to confront a medical consultant who is not amenable to appropriate, yet aggressive, interventions. It is our hope that by incorporating this scenario into clinical training, learners will be more confident in the correct stepwise diagnosis and treatment of NF, improve their interdisciplinary communication, and gain a greater sense of empowerment in dealing with consultants.

## Conclusions

This simulation scenario emphasizes the importance of including NF on a differential diagnosis as well as a structured approach to diagnosis and treatment. Additionally, learners are mentored to assertively advocate on behalf of their patient after inappropriate pushback from consultants. 
